# Dictionary learning compressed sensing reconstruction: pilot validation of accelerated echo planar J-resolved spectroscopic imaging in prostate cancer

**DOI:** 10.1007/s10334-022-01029-z

**Published:** 2022-07-23

**Authors:** Ajin Joy, Rajakumar Nagarajan, Andres Saucedo, Zohaib Iqbal, Manoj K. Sarma, Neil Wilson, Ely Felker, Robert E. Reiter, Steven S. Raman, M. Albert Thomas

**Affiliations:** 1 Radiological Sciences, University of California Los Angeles, Los Angeles, CA USA; 2 Urology, University of California Los Angeles, Los Angeles, CA USA

**Keywords:** Echo planar J-resolved spectroscopic imaging, Prostate cancer, Compressed sensing, Citrate, Myo-inositol

## Abstract

**Objectives:**

This study aimed at developing dictionary learning (DL) based compressed sensing (CS) reconstruction for randomly undersampled five-dimensional (5D) MR Spectroscopic Imaging (3D spatial + 2D spectral) data acquired in prostate cancer patients and healthy controls, and test its feasibility at 8x and 12x undersampling factors.

**Materials and methods:**

Prospectively undersampled 5D echo-planar J-resolved spectroscopic imaging (EP-JRESI) data were acquired in nine prostate cancer (PCa) patients and three healthy males. The 5D EP-JRESI data were reconstructed using DL and compared with gradient sparsity-based Total Variation (TV) and Perona-Malik (PM) methods. A hybrid reconstruction technique, Dictionary Learning-Total Variation (DLTV), was also designed to further improve the quality of reconstructed spectra.

**Results:**

The CS reconstruction of prospectively undersampled (8x and 12x) 5D EP-JRESI data acquired in prostate cancer and healthy subjects were performed using DL, DLTV, TV and PM. It is evident that the hybrid DLTV method can unambiguously resolve 2D J-resolved peaks including myo-inositol, citrate, creatine, spermine and choline.

**Conclusion:**

Improved reconstruction of the accelerated 5D EP-JRESI data was observed using the hybrid DLTV. Accelerated acquisition of in vivo 5D data with as low as 8.33% samples (12x) corresponds to a total scan time of 14 min as opposed to a fully sampled scan that needs a total duration of 2.4 h (TR = 1.2 s, 32 ${k}_{x}$×16 ${k}_{y}$×8 ${k}_{z}$, 512 ${t}_{2}$ and 64 ${t}_{1}$).

**Supplementary Information:**

The online version contains supplementary material available at 10.1007/s10334-022-01029-z.

## Introduction

Prostate cancer (PCa) is the second leading cause of cancer mortality and the most common cancer in men with an estimated 248,530 new cases of prostate cancer diagnosed in 2021 [1, 2]. PCa is typified by an unpredictable clinical course; therefore, early detection and accurate staging are not only paramount to identify patient-specific therapies but also for an early accurate assessment of aggressiveness of localized disease. Although serum prostatic-specific antigen (PSA) is a very sensitive test during early diagnosis, the specificity is low for cancer diagnosis [3, 4]. Suspected masses have been identified in the prostate using multi-parametric (mp) MRI, compared to transrectal ultrasound imaging, where mp-MRI includes T_2_-weighted imaging, diffusion-weighted images (DWI), apparent diffusion coefficients (ADC) maps derived from DWI and dynamic contrast-enhanced (DCE) MRI [5–8].

Three decades ago, ^1^H and ^31^P magnetic resonance spectroscopy (MRS) of the human prostate was first performed by Thomas et al. using a trans-rectal probe, demonstrating the ability of trans-rectal MRS to characterize proton and phosphorylated metabolites of normal, hyperplastic, and malignant prostates [9, 10]. Biochemical and histochemical studies have confirmed that the normal human prostate has a high level of citrate (Cit), which is greatly reduced, while choline (Ch) increases in malignant prostate [9, 11].

Magnetic Resonance Spectroscopic Imaging (MRSI), also known as Chemical Shift Imaging (CSI), facilitates the acquisition of spectral data from multiple regions of the prostate from either a selected volume of interest (VOI) or multiple slices [12, 13]. The total duration is very long since conventional MRSI uses phase-encoding steps to encode the spatial dimensions; however, elliptical weighted or average-weighted schemes have been used to shorten the total duration [14]. MRSI is a valuable technique for assessing the extent and aggressiveness of primary and recurrent PCa and the threshold (choline + creatine)/citrate images, when overlaid in color on T_2_W images, can estimate the spatial extent of PCa and benign prostatic hyperplasia (BPH). Kurhanewicz and co-workers assessed the efficacy of combined MRI and three-dimensional ^1^H MRSI in the detection and localization of PCa [15–17]. The aggressiveness of prostate cancer was evaluated by Scheenen and co-workers using MRSI [18]. Carroll and co-workers summarized findings from TRUS-guided biopsy, MRI and MRSI. In contrast to the TRUS detectability of only 35% of 114 patients showing lesions, 79 out of 114 patients showed an anatomic lesion characteristic of cancer using MRI and MRSI [19]. Using MRSI, DWI and MRSI + DWI, Hricak and co-workers recently developed statistically based rules for identifying cancer in the peripheral zone (PZ) [20]. Correlation of MRSI and MRI with molecular markers was demonstrated by Shukla-Dave et al. [21]. A multi-institutional prostate cancer study evaluated the incremental benefit of combined endorectal MRI and MRSI, as compared with endorectal MRI alone, for sextant localization of peripheral zone (PZ) prostate cancer [22]. Other multi-center studies conducted further validation of prostate cancer localization and aggressiveness [23, 24].

Even though k-space-weighted and average-weighted schemes have been used to shorten the total duration of MRSI, echo-planar spectroscopic imaging (EPSI) can further accelerate the total acquisition duration [25, 26]. Chen et al. showed high-speed 3 T spectroscopic imaging of prostate using flyback echo-planar encoding [27]. Adding a 2nd spectral dimension to MR spectroscopy helps to disperse the spectrum better. However, acquisition of MRSI after adding the 2nd spectral encoding can increase the total acquisition time significantly. Furuyama et al. applied compressed sensing (CS) reconstruction of accelerated J-resolved spectroscopic imaging acquisition in healthy human prostates [28]. Nagarajan et al. demonstrated the detection of Cit, Ch/Cr and Spm in prostate cancer using accelerated echo-planar J-resolved spectroscopic imaging (EP-JRESI) where a single-slice was localized and the efficiency of non-linear reconstruction using total variation (TV) and maximum entropy (MaxEnt) was compared [29].

Dictionary learning (DL) is another approach for adaptive sparse representation of signals in a CS framework [30]. In medical imaging, DL has been widely used for reconstruction in different areas like MRI, PET and CT [31–37]. DL in a CS reconstruction involves a process of learning dictionaries from training data and then generating a sparse representation using the learned dictionaries in an iterative manner [34]. The overcomplete set of basis functions learned by DL captures the underlying features of a signal devoid of noise, such that the learned set of basis functions can achieve a higher sparsity level for that particular signal [36, 37]. Ravishankar et al. proposed a DL-MRI scheme based on the K-SVD algorithm for learning the sparsifying transform for MRI reconstruction [34, 35]. It has since been one of the most popular methods to train dictionaries for MRI reconstruction among other methods like Method of Optimized Directions (MOD), Online Dictionary Learning (ODL) and Recursive Least Squares (RLS) [38–40].

In this work, we have used a five-dimensional (5D) MR Spectroscopic Imaging (MRSI) (3D spatial + 2D spectral) technique, which generates 2D spectra from multiple spatial locations covering a larger volume of prostate tissues in a single scan. One of the major challenges of this technique is the increased scan time, which can lead to higher motion related artifacts. A straightforward solution to this problem is an accelerated acquisition. However, with higher undersampling rates, the reconstruction performance also becomes a major contributing factor to the overall spectral quality. Therefore, the main aim of this study was to implement a more sophisticated reconstruction technique such as dictionary learning for MRSI and to assess its performance at higher undersampling factors compared with more conventional CS reconstruction techniques such as total variation, in order to test the feasibility of higher undersampling factors for prostate MRSI.

Hence, we have implemented and evaluated the performance of a hybrid DLTV reconstruction where DL iteratively trains dictionaries from a TV filtered data using K-SVD, on a set of non-uniformly sampled (NUS) 5D EP-JRESI data acquired in prostate cancer patients and healthy controls. Furthermore, we have compared the performance to using DL alone, TV and Perona-Malik (PM) reconstruction techniques [41, 42]. Undersampling rates of 8x and 12﻿x were imposed prospectively along two spatial (${k}_{y}$, ${k}_{z}$) and one spectral dimensions (${t}_{1}$) while the remaining spatial (${k}_{x}$) and spectral dimensions (${t}_{2}$) were fully encoded using an echo-planar readout. Multiple undersampling rates were retrospectively imposed on a fully encoded 5D EP-JRESI phantom data and reconstructions using DL, PM, TV and DLTV were investigated. Relative metabolite levels were quantified using the prior-knowledge based fitting (ProFit) algorithm [43].

## Materials and methods

### Human subjects

Nine PCa patients (mean age of 63 years) and three healthy males (mean age of 42.7 years) were investigated between May 2013 and June 2015. Gleason scores in the patients varied between 6 and 7. Prostate-specific antigen (PSA) levels among the PCa patients varied from 3 to 6.9 ng/mL. The PCa patients and healthy subjects were scanned with endorectal "receive" coil for patients and external phased-array "receive" coil for healthy using a 3 T Siemens (Siemens Medical Solution, Erlangen, Germany) MRI scanner. The protocol combining MRI and MRS was performed at least 8 weeks after the transrectal ultrasound-guided sextant biopsy in the PCa patients. The entire protocol was approved by the Institutional Review Board (IRB), and informed consent was obtained from each subject.

### MRI and MRSI

All patients and healthy subjects were imaged in the supine (feet-first) position. Axial images were oriented to be perpendicular to the long axis of the prostate, which was guided by the sagittal images. Axial, coronal, and sagittal T_2_-weighted (T_2_W) turbo spin-echo images were recorded using the following parameters: repetition time/echo time (TR/TE), 3850–4200/96–101 ms; slice thickness, 3 mm; field of view, 20 × 20 cm^2^; echo train length (ETL), 13 and data matrix, 320 × 256.

**Fig. 1:**
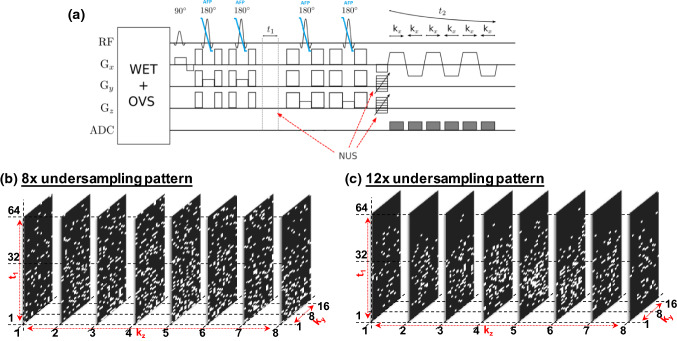
**a** The pulse sequence diagram for 5D NUS EP-JRESI. **b** An example of non-uniform sampling pattern based on exponentially decaying probability density for 8x undersampling. White and black colors represent acquired and unacquired locations in the k-space. **c** An example of non-uniform sampling pattern based on exponentially decaying probability density for 12x undersampling. White and black colors represent acquired and unacquired locations in the k-space

A maximum echo-based 5D EP-JRESI sequence [44] as shown in Fig. 1a was used and the volume-of-interest (VOI) was localized using a semi-LASER PRESS module with five slice-selective radio-frequency (RF) pulses, the first 90^0^ RF pulse followed by two pairs of adiabatic full passage RF pulses [45] (Fig. 1a). The acquisition parameters for the 5D EP-JRESI were: TR/TE/Avg = 1200 ms/41 ms/1, 16 $${k}_{y}$$_*_8 $${k}_{z}$$ phase encoding steps, field of view (FOV) = 16 × 16 × 12 cm^3^, 512 complex points (t_2_) with an $${F}_{2}$$ spectral bandwidth of 1190 Hz along the detected spectral dimension. For the indirect (2nd) dimension ($${F}_{1}$$), 64 $${t}_{1}$$ increments with a spectral bandwidth of ± 250 Hz were used. The spatial resolution in terms of cubic voxels with nominal dimensions were calculated from the FOV and the matrix size as 1 × 1 × 1.5cm^3^ [46]. Since the EPSI readout simultaneously acquires one spatially encoded dimension ($${k}_{x}$$) and one temporal dimension ($${t}_{2}$$), we imposed nonuniform undersampling (NUS) along the remaining ($${k}_{y}$$-$${k}_{z}$$-$${t}_{1})$$ dimensions. NUS rates of 8x and12x were imposed as shown in Fig. 1b, c. Combination of spatial and spectral dimensions in the reconstruction is expected to introduce more sparsity than the undersampled spatial dimensions alone. Two sets of data were collected, one water suppressed scan (WS) with a total scan time of 21 min (8x) and 14 min (12x), and a second non-water suppressed scan (NWS) using one average and one $${t}_{1}$$ increment (approx. 2.6 min). The NWS scan was used for eddy current phase correction and coil combinations. WET-suppression was used for the global suppression of water [47]. The average full width half maximum (FWHM) of the water peak was $$30.13\pm 9.29Hz$$ over the localized VOI including the cancer and non-cancer locations.

## Phantom

A fully sampled 5D EP-JRESI data of a home-made prostate phantom was acquired with the body coil and was retrospectively undersampled at different rates to compare the reconstruction performance of DL, DLTV, TV and PM. The total time for acquiring the fully sampled 5D EP-JRESI scan (TR of 1.5 s, 32 ${k}_{x}$_,_ 16 ${k}_{y}$, 8 ${k}_{z}$, 512 ${t}_{2}$, 64 ${t}_{1}$) was 3 h, 24 min and 48 s. The fully sampled data were retrospectively undersampled at 2x, 4x, 8x, 12x and 16x levels and a non-uniform sampling pattern based on an exponentially decaying probability density was used [44], as shown in Fig. 1b, c, where b and c represent 8x and 12x accelerations, respectively. A 500-ml prostate phantom was prepared with the following concentrations: citrate (Cit, 50 mM), creatine (Cr, 5 mM), choline (Ch, 1 mM), spermine (Spm, 6 mM), myo-inositol (mI, 10 mM), phosphocholine (PCh, 2 mM), taurine (Tau, 3 mM), glutamate (Glu, 4 mM), glutamine (Gln, 2.5 mM) and scyllo-inositol (sI, 0.8 mM). 50 mM sodium formate and 1 mM DSS (3-(trimethylsilyl)-1-propanesulfonic acid sodium salt) were also added to the prostate phantom to standardize chemical shifts.

## Data analysis and working principles of CS techniques in MRSI

Non-linear reconstructions were performed on the non-uniformly undersampled data using DL, DLTV, TV and PM. Random sampling causes the undersampling artifacts to appear noise-like. When the signal to be reconstructed has a sparse representation, CS theory holds that the artifact free signal can be recovered by approximating it using a few of its sparse coefficients in a non-linear fashion [48–50]. The presence of aliasing artifacts decreases the sparsity of the data and, therefore, the sparse approximation enables recovery of the underlying signal devoid of artifacts. While TV and PM reconstruction techniques assume data sparsity in the finite difference representation, DL learns an overcomplete set of basis functions, or dictionary, that can represent the data in sparse form. DLTV on the other hand assumes sparsity with respect to both the finite difference representation and the learned basis.

## Perona-Malik and total variation

The feasibility of PM and TV based CS reconstruction is due to the fact that the MRSI data has sparse gradients. Please note that the term ‘gradient’ from here on refers to the directional change in the intensity of MRSI data in the image/spectral domain and is not to be confused with the variation in the magnetic field. Gradients with larger magnitude are generally representative of the signal of interest as compared to the lower magnitudes which usually represent noise. Hence, both PM and TV attempt to separate the signal from undersampling artifacts based on its gradient magnitude. While TV denoises the signal by minimizing the ${l}_{1}$ norm of the gradients, PM achieves the same by minimizing the Lorentzian error norm [51, 52]. The conventional PM denoises the signal $m$ by diffusing it over a small time $\partial t$ as

1 $$\partial m =\zeta div\left(g\left(\left|\nabla m\right|\right)\nabla m\right).$$

where $\zeta$ is a regularization parameter (step-size) controlling the strength of denoising and $div$ is the divergence operator, and $g\left(\left|\nabla m\right|\right)$ is the diffusivity function. The diffusivity function defines a spatially varying weight that controls the extent of smoothing such that stronger smoothing is performed in the regions where the gradient magnitudes are smaller. The Lorentzian error norm is minimized when the choice of diffusivity function is

2 $$g\left(\left|\nabla m\right|\right)=\frac{1}{1+{\left(\left|\nabla m\right|/\alpha \right)}^{2}}$$

where $\alpha$ is a gradient threshold parameter that separates the gradient magnitudes of signal from noise. The formulation becomes equivalent to TV when $g\left(\left|\nabla m\right|\right)=1/\left|\nabla m\right|$.

In a compressed sensing framework, PM operates by performing denoising followed by a data consistency step in each iteration so as to minimize a cost function of the form

3 $$\underset{m}{\mathrm{min}}\;lor\left(\left|\nabla m\right|\right)\mathrm{ s}.\mathrm{t}. {\Vert {F}_{u}m-s\Vert }_{2}^{2}<\epsilon$$

where $lor\left(\left|\nabla m\right|\right)={\sum }_{i}\mathrm{log}\left(1+\frac{1}{2}{\left(\frac{\left|\nabla m\right|}{\sigma }\right)}^{2}\right)$, ${\sigma }^{2}=\frac{{\alpha }^{2}}{2}$ and $i$ is the number of elements in $m$ [41, 51]. The term $s$ is the acquired undersampled k-space data with zeros at unacquired locations. ${F}_{u}$ computes the forward and inverse Fourier transforms of $m$ in image and temporal domains, respectively, and then sets the values at unacquired locations of k-space as zeros. The constraint in Eqn. [3] ensures that the deviation of reconstructed data from the acquired data at sampled locations is restricted.

A good value of $\alpha$ can be estimated from the data as the mean/median absolute deviation of the gradients or using the noise estimator described by Canny [53]. We used the mean absolute deviation (MAD) as reported in [41] to estimate $\alpha$ in this work. While TV becomes sensitive to the choice of $\zeta$, the presence of $\alpha$ helps PM to be stable over a wider range of values of $\zeta$, with a typical choice being $\zeta =0.1$ for PM [41]. This gives a good compromise between reconstruction quality and reconstruction time. Smaller values of $\zeta$ generally do not give a significant improvement in reconstruction quality but increases the number of iterations required for convergence [41].

The TV based reconstruction is formulated as a cost function minimization of the form

4 $$\underset{m}{\mathrm{min }}{\left|\nabla m\right|}_{1}\mathrm{ s}.\mathrm{t}. {\Vert {F}_{u}m-s\Vert }_{2}^{2}<\epsilon$$

Due to the better sensitivity to the regularization parameter when solved in a modified Split-Bregman framework as reported in [28, 29], we used the Split-Bregman algorithm for CS-TV reconstruction in this work, as described in [44]. CS-PM was implemented as described in [41]. In both cases, the choice of $\epsilon$ controls the fidelity with values at sampled locations of k-space. Larger values of $\epsilon$ allows the reconstruction to deviate more from the known k-space samples at acquired locations and helps to minimize the noise by smoothing. A strict data fidelity constraint of ${F}_{u}m=s$ can lead to noisy reconstructions when the noise level in the acquired data are high. Ideally, $\epsilon$ should be set at the level of noise in the acquired data. In the case of PM, adaptation of $\alpha$ helps to minimize noise in reconstructed data. Therefore, a strict data fidelity is used and $\alpha$ is adaptively chosen in each iteration using the MAD of the gradients.

## Dictionary learning and hybrid dictionary learning- total variation

The DL-based CS reconstruction learns an overcomplete set of basis functions, or dictionary, that captures the underlying features of a signal devoid of noise, such that the learned dictionary can achieve a higher sparsity level for the particular signal of interest [35, 36]. When the aliasing due to non-uniform undersampling has noise-like properties, trained dictionaries become capable of removing the aliasing artifacts, and thereby reconstruct the underlying signal.

One of the drawbacks of using a fixed basis (as in the case of finite difference representation) is that such a basis might not be universally optimal for all datasets [35]. Since DL works by learning a basis which is specific to the data under reconstruction, it has the potential to find a better sparse representation for it, which can in turn improve the quality of the reconstructed data in a CS framework.

Basic elements in a sparsifying dictionary are called atoms, whose linear combinations can represent a given signal in sparse form. We use one of the most popular approaches to train such dictionaries, called the K-SVD algorithm [34]. In this method, the dictionary is updated iteratively atom by atom. In each iteration, sparse coefficients of the signal are updated based on the current estimate of the dictionary and then the dictionary atoms are updated to best fit the current sparse representation of the signal.

The algorithm was implemented as described in [54], based on the MATLAB codes for the same, publicly available at [55]. DL is known to have a slow reconstruction speed as compared to TV and PM, due to the training of dictionaries in each iteration. Therefore, we have used the acceleration technique of fast iterative soft thresholding algorithm (FISTA) to accelerate the reconstruction [56].

**Fig. 2:**
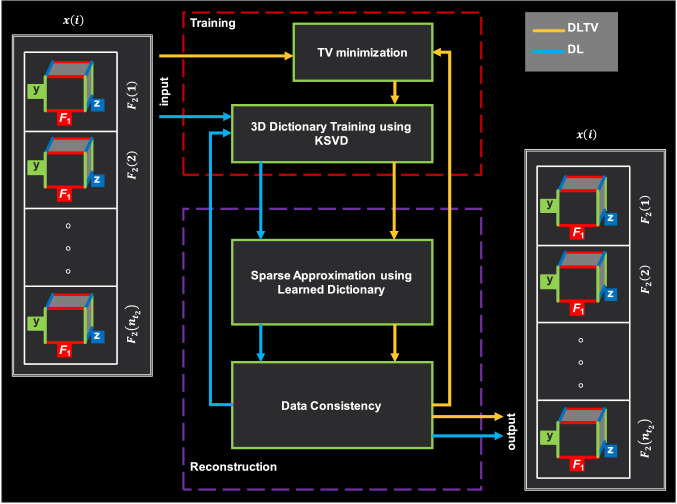
Comparison of DL and DLTV workflow. Yellow and blue colored arrows represent DL and DLTV workflow, respectively. $y$, $z$ and ${F}_{1}$ represents the Fourier transform of non-uniformly undersampled phase-encoding dimensions and indirect spectral dimension ${t}_{1}$ after filling the missing samples with zeros. ${F}_{2}$ and $x$ are the Fourier transforms of fully sampled ${t}_{2}$ and ${k}_{x}$ dimensions

Further acceleration was achieved by operating the 3D-DL reconstruction in a customized 3D space formed by stacking the direct spectral dimension (${F}_{2}$) as shown in the work-flow in Fig. 2. This approach accelerates the reconstruction by training a single dictionary for ${F}_{2}$, instead of having to learn separate dictionaries for each point along ${F}_{2}$. The variables $y$, $z$ and ${F}_{1}$ represent the Fourier transforms of two phase encoded spatial dimensions ${k}_{y}$ and ${k}_{z}$, and indirect spectral dimension ${t}_{1}$. $x$ represents the Fourier transform of the fully sampled readout dimension ${k}_{x}$.

The acquired data were first zero-filled and Fourier transformed before rearranging into ${n}_{x}$ groups as shown in Fig. 2. Overlapping 3D blocks were then extracted from a regular grid on the real and imaginary parts of the readout points, $x\left(i\right) \forall i=\mathrm{1,2},\dots ,{n}_{x}$, which were used by the K-SVD algorithm to train the dictionary [54]. Once the dictionary was learned, an orthogonal matching pursuit (OMP) algorithm was used to sparsely code the real and imaginary parts of $x\left(i\right)$ independently [57]. Then the data consistency step was enforced by correcting the values at locations of acquired k-space samples in the reconstructed data. This process was then repeated to find the set of updated dictionaries and the subsequent set of sparse coefficients for the data, followed by a data consistency step in each iteration until convergence.

The associated cost function minimization at a point $x\left(i\right) \;\forall i=\mathrm{1,2},\dots ,{n}_{x}$ is of the form

5 $$\underset{\begin{array}{c}D,{m}_{x(i)},\\ {\rho }_{\mathcal{R}}, {\rho }_{\mathcal{I}}\end{array}}{\mathrm{min}}\sum_{j}\left({\Vert {\rho }_{\mathcal{R},j}\Vert }_{0}+{\Vert {\rho }_{\mathcal{I},j}\Vert }_{0}\right)+\nu {\Vert {F}_{u}{m}_{x\left(i\right)}{-s}_{x\left(i\right)}\Vert }_{2}^{2} \mathrm{s}.\mathrm{t}. \left\{\begin{array}{c}{\Vert D{\rho }_{\mathcal{R},j}-{\mathbb{R}}_{j}\mathcal{R}\left({m}_{x\left(i\right)}\right)\Vert }_{2}^{2}<\epsilon \\ {\Vert D{\rho }_{\mathcal{I},j}-{\mathbb{R}}_{j}\mathcal{I}\left({m}_{x\left(i\right)}\right)\Vert }_{2}^{2}<\epsilon \end{array}\right\}\forall j$$

where ${s}_{x(i)}$ and ${m}_{x(i)}$ are the acquired and reconstructed custom-3D k-space data, respectively, at every point in $x$. $\nu$ controls the consistency of reconstructed data with acquired k-space samples. $D$ is a real valued dictionary that can sparsely represent both real and imaginary components of ${s}_{x(i)}$, and is adaptively learned. ${\mathbb{R}}$ is an operator that extracts 3D blocks from the customized 3D space and $j$ is the patch number. $\rho$ is the sparse representation of the extracted blocks. $\mathcal{R}$ and $\mathcal{I}$ denote the real and imaginary components of the complex data, respectively.

The DLTV is a combination of DL and TV that sparsely approximates the data using both learned dictionaries and finite difference representations, thereby further increasing the sparsity of the data [58].

This modifies the cost function in Eqn. [5] as

$$\underset{\begin{array}{c}D,{m}_{x\left(i\right)},\\ {\rho }_{\mathcal{R}}, {\rho }_{\mathcal{I}}\end{array}}{\mathrm{Min}}\sum_{j}\left({\Vert {\rho }_{\mathcal{R},j}\Vert }_{0}+{\Vert {\rho }_{\mathcal{I},j}\Vert }_{0}\right)+{\mu \left|\nabla {m}_{x\left(i\right)}\right|}_{1}+\nu {\Vert {F}_{u}{m}_{x\left(i\right)}-{s}_{x\left(i\right)}\Vert }_{2}^{2}$$

6 $$\mathrm{s}.\mathrm{t}. \left\{\begin{array}{c}{\Vert D{\rho }_{\mathcal{R},j}-{\mathbb{R}}_{i}\mathcal{R}\left({m}_{x\left(i\right)}\right)\Vert }_{2}^{2}<\epsilon \\ {\Vert D{\rho }_{\mathcal{I},j}-{\mathbb{R}}_{i}\mathcal{I}\left({m}_{x\left(i\right)}\right)\Vert }_{2}^{2}<\epsilon \end{array}\right\}\forall j$$

where $\mu$ controls the gradient sparsity. A comparison of the DL and DLTV workflows is shown by the yellow and blue arrows in Fig. 2. The main difference in the workflow is that the DLTV trains dictionaries from TV filtered data instead of directly learning the dictionaries from the zero-filled and Fourier transformed data in DL.

The parameters of reconstruction were empirically chosen for an efficient overall reconstruction of undersampled phantom as follows: A fully sampled phantom data was retrospectively undersampled at different sampling rates and reconstructed using DLTV. The DLTV parameters were then chosen for each undersampling level to minimize the error in reconstruction and then used for in-vivo reconstruction. With the acceleration scheme used in this paper for DL, it is observed that DL converges in around half the number of iterations as that of TV, despite the fact each iteration of DL was much slower than that of TV. Hence, the DLTV reconstruction framework was designed to keep this factor in consideration, such that the overall convergence rate in terms of the number of iterations is relatively the same for both TV and DL in DLTV.

## Error metric evaluation

The following normalized root mean squared error (nRMSE) measure is used to evaluate the performance of the different reconstruction techniques compared in this work:

7 $$nRMSE=\frac{100}{\sqrt{{N}_{s}}}\times \frac{{\Vert {data}_{R}-{data}_{GT}\Vert }_{2}}{{\Vert {data}_{GT}\Vert }_{2}}$$

where ${data}_{GT}$ is the fully sampled ground truth, ${data}_{R}$ is the reconstructed data and ${N}_{s}$ is the number of elements in $dat{a}_{R}$.

Furthermore, the 2D J-resolved spectra were individually assessed both qualitatively (by inspecting the difference in recovered signal intensity) and quantitatively (by prior-knowledge fitting of the spectra using various metabolites which are reported in prostate tissues) to determine the quality of each reconstruction, particularly the ability of each method to recover the diagonal peaks and cross-peaks of the main prostate metabolites.

The fitting of metabolites is based on the ProFit algorithm that fits the set of simulated spectra and measures the quality of the fit by comparing creatine 3.9 (Cr3.9) to creatine 3.0 (Cr3.0) ratios. This ratio should ideally be 1 since the number of protons are already considered in the basis-set creation for Cr3.9 and Cr3.0. Results with higher Cr3.9/Cr3.0 ratios can be excluded due to the lack of acceptable fitting.

## Results

The reconstruction performances of PM, TV, DL and DLTV were studied using retrospectively undersampled phantom data and prospectively undersampled 5D EP-JRESI as described in the following sub-sections. DLTV reconstructions of 4D EP-JRESI datasets are also included in the Supplementary section.

## Phantom

**Fig. 3:**
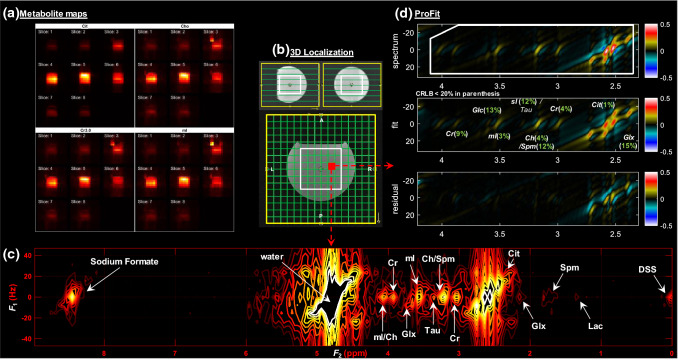
**a** metabolite maps of citrate at 2.5 ppm, creatine at 3 ppm, choline/spermine at 3.2 ppm and myo-Inositol at 3.5 ppm. **b** 3D localization images. **c** labeled contour plot of J-resolved 2D spectrum from a single voxel location in the fully sampled prostate phantom. **d** Profit based fit of the spectrum and the residual. CRLB < 20% is shown in the insert of the fit inside parenthesis

Figure 3c shows a typical 2D J-resolved spectrum, generated from a voxel of phantom data with metabolites at physiological concentrations acquired using the fully sampled 5D EP-JRESI sequence. The corresponding volume localization images (Fig. 3b) and metabolite maps (Fig. 3a) indicate that 4 out of 8 slices are within the volume of interest (VOI) represented by the white box, either fully or partially. The metabolite maps were obtained by integrating the corresponding 2D metabolite peaks.

Figure 3d shows the fit and residual of the spectrum using ProFit, and the respective Cramér Rao lower bounds (CRLB) of the fit for each metabolite. The fit error is minimized within the spectral range indicated by the white box in the spectrum. The CRLB is used an indicator of the minimum error for estimated parameters, where lower values indicate a more reliable fit. CRLB < 20% are shown in parentheses including Cr, Spm, Cit, Glx(Glu + Glx), tCh(Ch + pCh), mI and sI. The CRLB for Tau was higher due to significant overlap with other metabolites. mI/Ch, Ch/Spm and sI/Tau in this figure as well as the in all the remaining figures indicate the overlapping resonances and not their ratios.

This fully sampled prostate phantom data was retrospectively undersampled at 2x, 4x, 8x, 12x and 16x accelerations corresponding to 50%, 25%, 12.5%, 8.33% and 6.25% acquired k-space samples, respectively. Reconstruction was then done using DL, TV, DLTV and PM at each undersampling factor. The reconstruction error relative to the fully sampled data (ground truth) was measured using nRMSE as defined in eqn. [7] and the values are listed in Table 1.

**Table 1:**
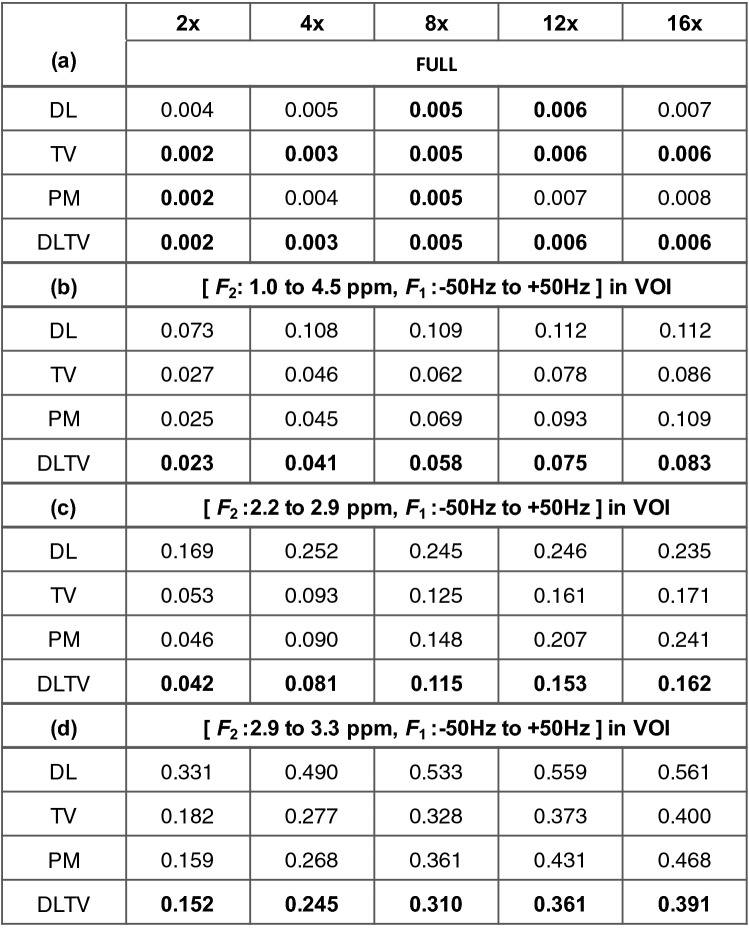
nRMSE comparison of PM, TV, DL and DLTV reconstruction of prostate phantom data retrospectively undersampled at 2x, 4x, 8x, 12x and 16x

The bolded numbers represent lowest nRMSE measures. (a) nRMSE in the full range of spectrum, across all voxels. (b) nRMSE in the voxels within VOI in the range of 1 to 4.5 ppm along ${F}_{2}$ and -50 to + 50 Hz along ${F}_{1}$ dimensions. (c) nRMSE values within VOI in the range of 2.2 to 2.9 ppm along ${F}_{2}$ and -50 to + 50 Hz along ${F}_{1}$ dimension. (d) nRMSE values within VOI in the range of 2.9 to 3.3 ppm along ${F}_{2}$ and − 50 to + 50 Hz along ${F}_{1}$ dimension

Section (a) in the table shows how well each method reconstructs the data within the entire ${F}_{2}$-${F}_{1}$ plane. Section (b) shows the error in reconstruction within the VOI in the range of 1 to 4.5 ppm and -50 to + 50 Hz in ${F}_{2}$ and ${F}_{1}$ dimensions, respectively. The nRMSE values in section (a) show similar performance for DL, TV, PM and DLTV at all acceleration factors. In section (b), TV, PM and DLTV show similar performance from 2x to 8x accelerations. However, on closer observation, we can see that the DLTV has the lowest nRMSE values (bolded numbers). Both TV and DLTV show lower nRMSE values compared to PM and DL at 12x and 16x accelerations. The values for DL are higher than PM and TV at lower undersampling factors, and these values become comparable at higher undersampling levels. Sections (c) and (d) in the table report the nRMSE values in the range of 2.2 to 2.9 ppm containing the citrate peak, and from 2.9 to 3.3 ppm containing peaks due to creatine, choline, spermine and taurine. While the nRMSE measures across different acceleration factors follow a similar trend as previously mentioned, the error measures from 2.9 to 3.3 ppm appear to be higher than those from 2.2 to 2.9 ppm. The fact that the SNR in the former region is lower compared to the latter suggests an overall better reconstruction performance in the regions with high SNR.

## In vivo

**Fig. 4:**
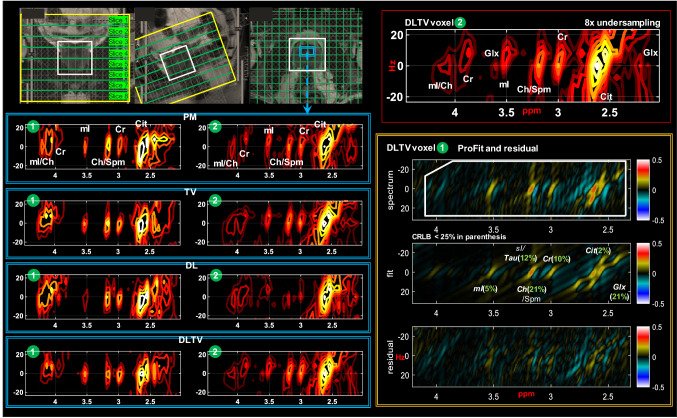
Reconstruction results of a prospectively undersampled (8x) 5D EP-JRESI data acquired in a 26-year-old healthy volunteer. Localization images of sagittal, coronal and axial planes are shown in the top-left panel. Bottom-left panel shows the distribution of spectra (${F}_{2}$: 2 to 4.5 ppm, ${F}_{1}$:  − 25 to + 25 Hz) within the blue bounding box in the VOI (white bounding box), reconstructed using PM, TV, DL and DLTV. The 2D spectra show different metabolites including citrate, creatine, choline, spermine, myo-Inositol and Glu/Gln. Individual voxels are labeled using green circles. An enlarged view of DLTV voxel 3 with labeled metabolites is shown on the top-right panel. Profit based fit and residual of the spectrum in voxel 1 are shown in bottom-right panel. CRLB < 25% is shown in the insert of the fit

**Fig. 5:**
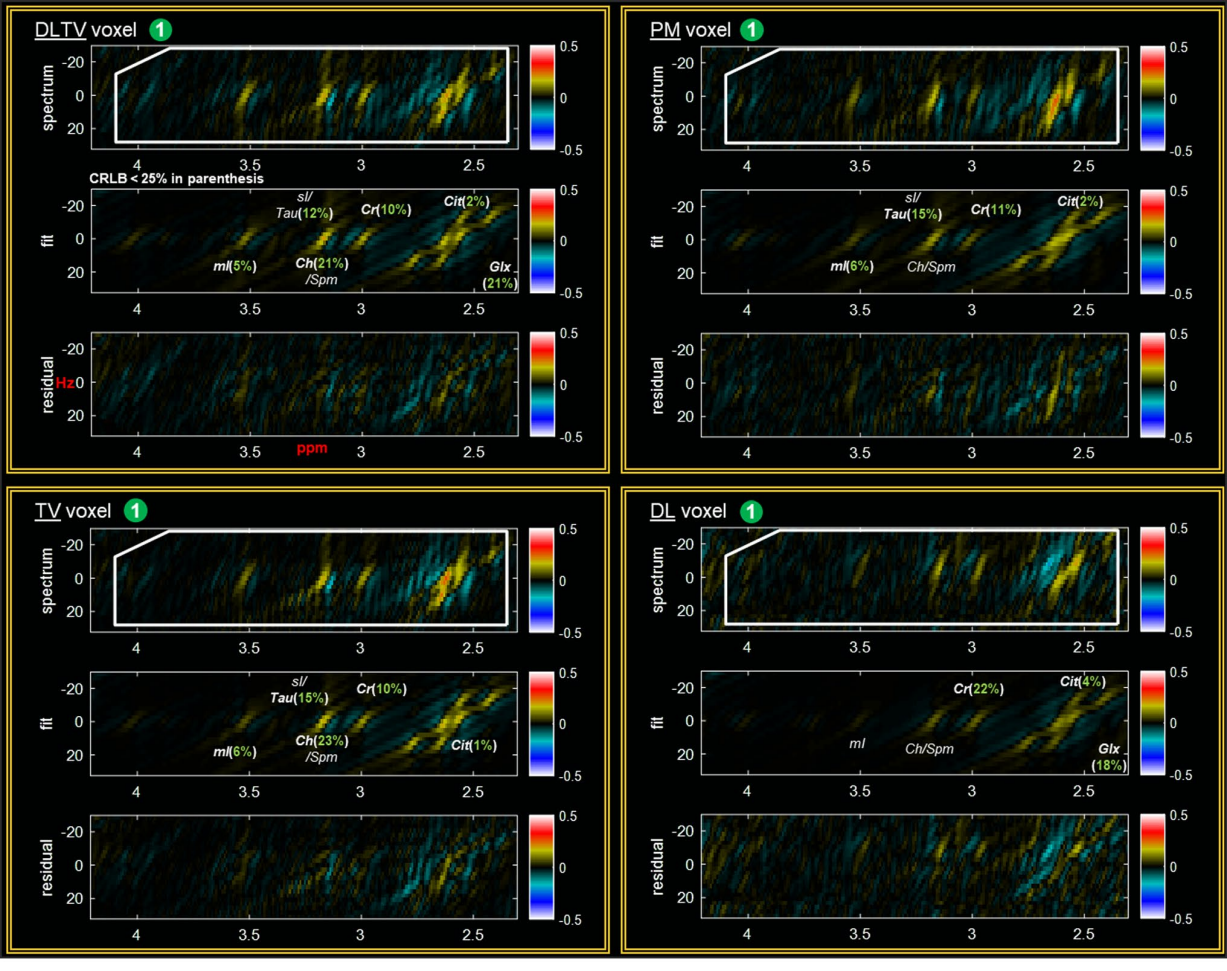
Profit based fit and residual of the spectrum in voxel 1 of Fig. 4 for DLTV, TV, PM and DL reconstructions. CRLB < 25% is shown in the insert of the fit

Figure 4 shows the reconstruction of a prospectively undersampled (8x) in-vivo prostate scan of a 26-year-old healthy volunteer using PM, TV, DL and DLTV. Localization images of sagittal, coronal and axial planes are shown in the top panel. The bottom-left panel shows the distribution of spectra (${F}_{2}$: 2 to 4.5 ppm, ${F}_{1}$: − 25 to + 25 Hz) within the blue box inside the VOI. The spectra show different metabolites described in Fig. 3, including Cit (2.6 ppm), Cr (3 ppm, 3.9 ppm), Ch (3.2 ppm), mI (3.5 ppm) and Glx (2.2–2.4 ppm). Individual voxels are labeled in green circles. A ProFit based fitting for quantitative analysis of voxel 1 reconstructed using DLTV is shown on the bottom-right panel with CRLB values < 25% in parentheses. A comparison between different reconstructions of voxel 1 using ProFit fitting is shown in Figs. 5. While the metabolite ratios were not significantly different between these reconstruction methods, better reliability of fitting based on CRLB for more number of metabolites was observed with DLTV.

**Fig. 6:**
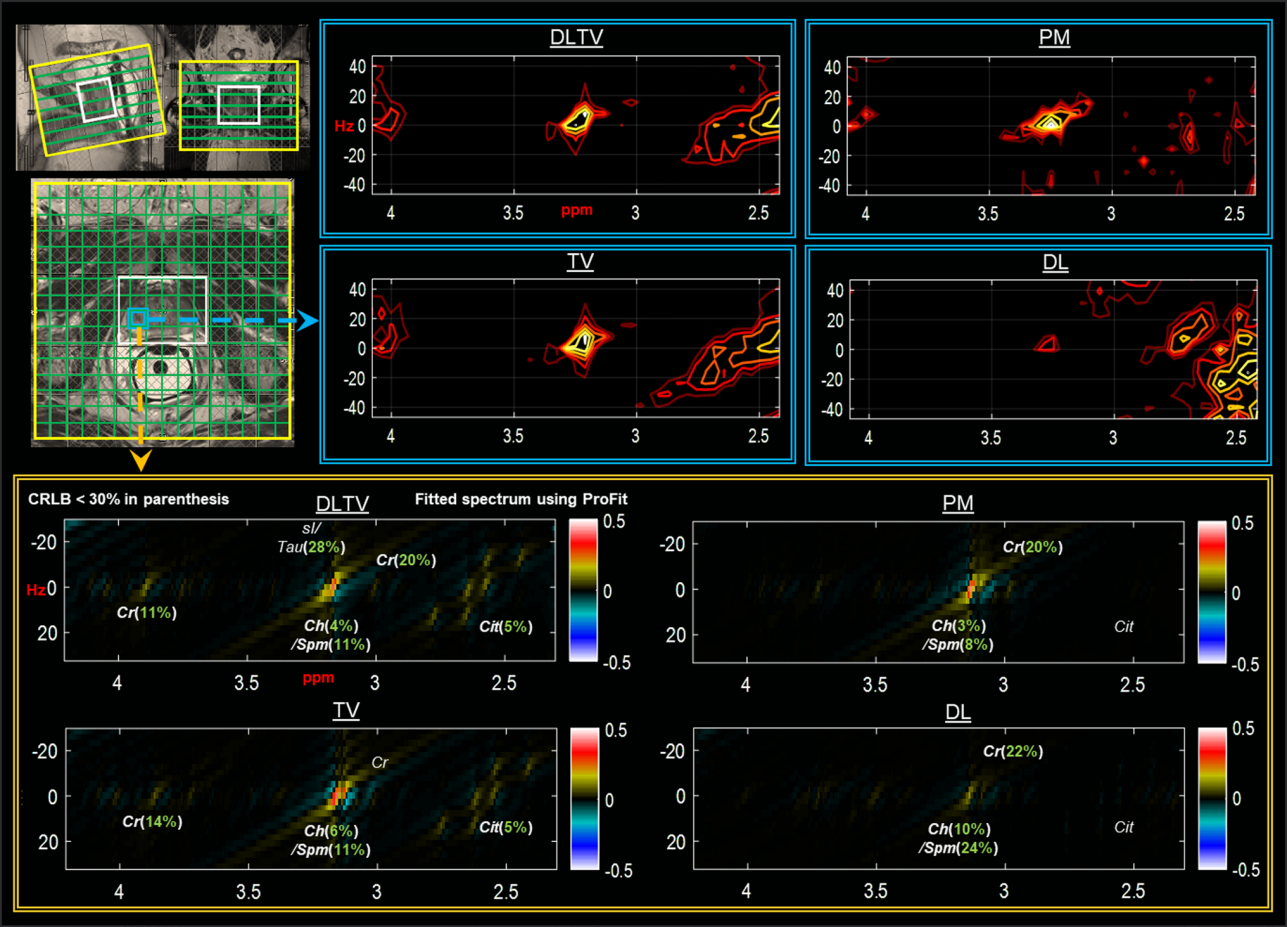
Reconstruction results of a prospectively undersampled (8x) 5D EP-JRESI data acquired in a 74-year-old patient in-vivo 5D EP-JRESI prostate data (Gleason score of 3 + 3). Localization images of sagittal, coronal and axial planes are shown in the top panel. The DLTV, TV, PM and DL reconstruction results of a voxel identified as malignant by biopsy are shown on the right side of localization images. Profit based fit of this voxel for different reconstructions are shown below the localization images. CRLB < 30% is shown in the insert of the fit

**Fig. 7:**
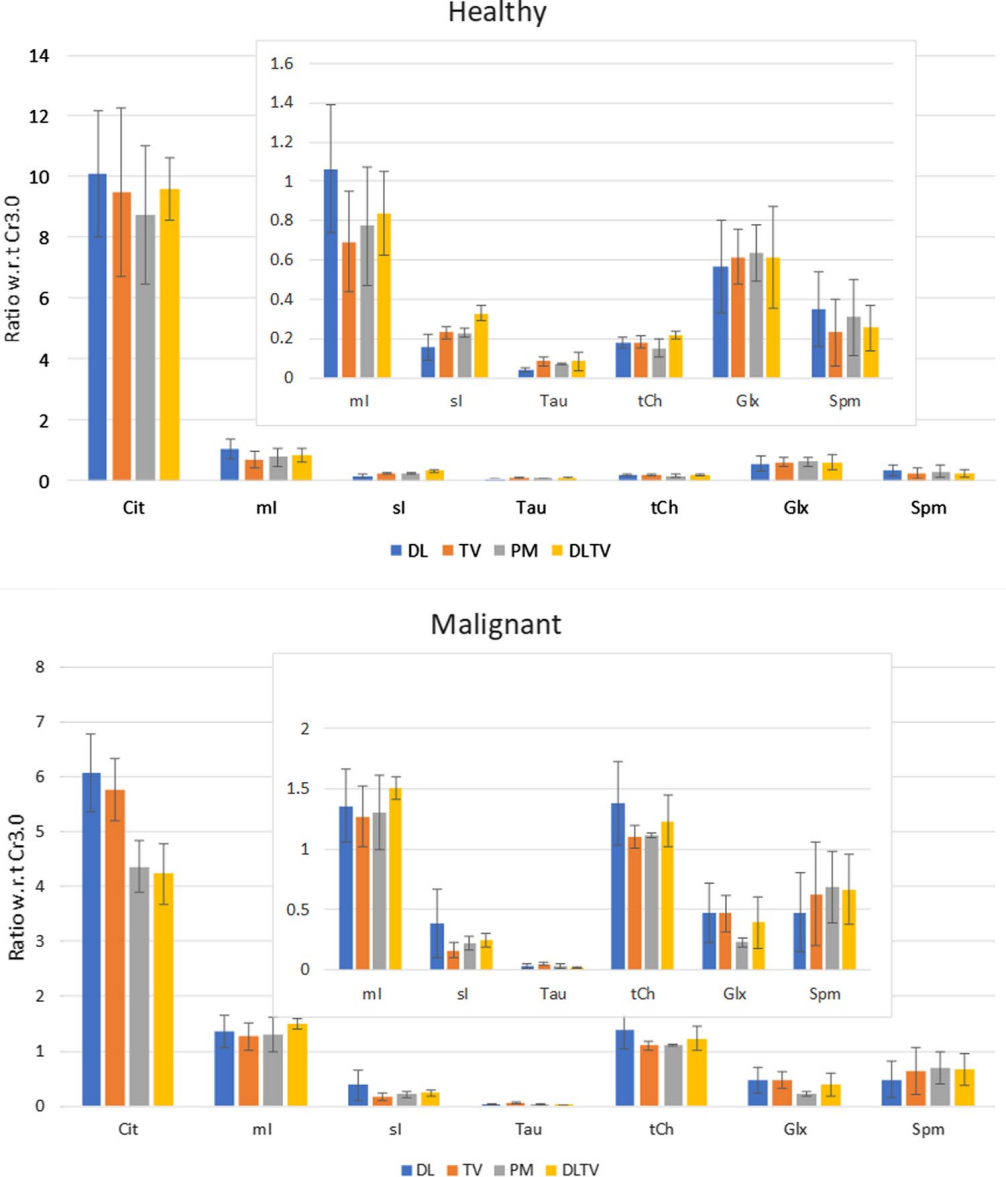
Average metabolite ratios of healthy and malignant voxels with respect to Creatine 3.0. Healthy voxels were selected from healthy volunteers. The malignant voxels were selected based on the locations identified by biopsy in patients

ProFit fitted spectra from another malignant location in a 74-year-old patient (Gleason score of 3 + 3), reconstructed using different approaches, are shown in Fig. 6. Better fit of metabolites in terms of CRLB was achieved with DLTV reconstruction. In addition to Cit (5%), Ch (4%), Spm (11%), Cr3.0 (20%) and Cr3.9 (11%), mI and Tau were fitted with higher CRLB (> 25%). The results of quantitation comparing metabolite ratios in healthy and malignant locations are shown in Fig. 7. Healthy voxels were selected from healthy volunteers and the malignant voxels were selected based on the anatomical images combined with results from biopsy in each patient. Even though trends of increased Ch/Cr, mI/Cr, and decreased Glx/Cr, Cit/Cr and sI/Cr ratios agreed with previously reported ex-vivo HR-MAS studies [59, 60], overestimation of Spm/Cr ratio was observed in malignant lesions [43].

**Fig. 8:**
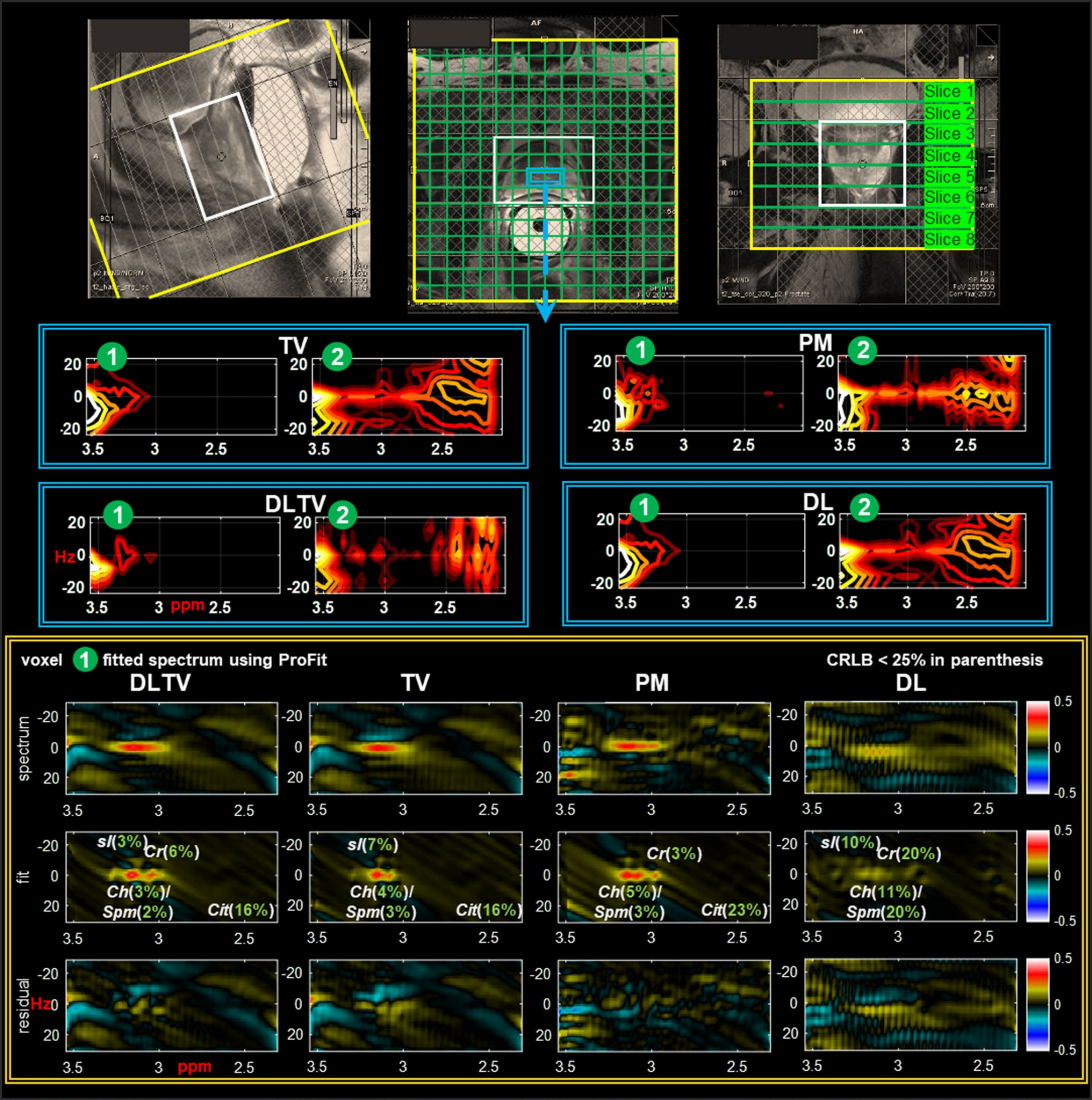
Reconstruction results of a prospectively undersampled (12x) 5D EP-JRESI data acquired in a 48-year-old patient in-vivo (Gleason score of 4 + 3). Localization images of sagittal, coronal and axial planes are shown in the top panel. Panels in the middle show the distribution of spectra (${F}_{2}$: 2 to 3.6 ppm, ${F}_{1}$: -25 to + 25 Hz) within the blue bounding box in VOI (white bounding box), reconstructed using PM, TV, DL and DLTV. Individual voxels are labeled using green circles. Profit based fit of voxel 1 and the residual are shown in the bottom most panel within yellow bounding box

Figure 8 shows the reconstruction performance at 12x acceleration. Only 8.33% k-space samples were collected from a 48-year-old PCa patient (Gleason score of 4 + 3) using an endorectal probe and the remaining samples were estimated using PM, TV, DL and DLTV. The J-resolved 2D spectra (${F}_{2}$: 2 to 3.6 ppm, ${F}_{1}$: -25 to + 25 Hz) from voxels within the blue box are shown below the localization images, followed by the ProFit fitted spectra of voxel 1. Depleted citrate is seen in voxel 1 indicates a cancerous location. CRLB shows better fit of metabolites using ProFit when reconstructed using DLTV with Cit (16%), Ch (3%), Spm (2%), Cr3.0 (6%) and sI (3%).

Extension of the proposed reconstruction using DLTV for the prospectively undersampled 4D-EPJRESI data is straightforward by using 2D dictionaries instead of 3D [35, 54]. Additional figures showing the results of DLTV reconstruction of undersampled 4D EP-JRESI datasets are included in the supplementary section, comparing the reconstruction performance of TV and DLTV,as opposed to TV and maximum entropy reported in [29].

## Discussion

The application of DL and DLTV reconstruction techniques to non-uniformly undersampled 5D EP-JRESI data was studied and evaluated in comparison with the gradient sparsity-based techniques of PM and TV, using both phantom and in-vivo datasets at various acceleration factors. The theory of CS requires that the data have an associated sparse representation to reconstruct it from the non-uniform samples collected at a sub-Nyquist rate. Higher sparsity helps to better recover the data in a CS framework. Therefore, we have compared finite difference based sparse representation, DL based sparse representation, and a combination of both (DLTV), for CS reconstruction at undersampling factors ranging from 2x to 16x.

While the chemical shift misregistration is expected to be reduced with the semi-LASER localization, a less effective outer volume suppression or the effect of Gibbs ringing due to low spatial resolution can cause contamination of voxels. The effect of Gibbs ringing can be reduced by increasing the spatial resolution, but it will then reduce the sensitivity of measurement and hence lead to a trade off with SNR. Similarly, one could apply k-space filters like Fermi or Hamming filter functions [13] to reduce the ringing effect during post processing at the cost of blurring (increased voxel size). The figures shown do not include the application of such filters.

The ability of resolving 2D J-resolved peaks including mI, sI, Tau, Cit, Cr, Spm, Ch and Glx, in multiple voxels, as well as the ability to quantify individual metabolite ratios with respect to Cr3.0 using ProFit is evident in Figs. 3–8. This is in contrast to a conventional 1D MRS analysis which uses (Cho + Cr)/Cit ratio or (Cho + Spm + Cr)/Cit ratio [61].

nRMSE is a useful metric to measure the difference in performance between different reconstruction techniques in retrospectively undersampled phantom. Table 1 shows better performance of DLTV in terms of nRMSE from 2x to 16x undersampling levels. In the absence of ground truth as in the case of in vivo data, a comparison based on metabolite ratios or reliability of ProFit based fitting in terms of CRLB can be made. Since the average difference in metabolite ratios were in a similar range across all four reconstruction techniques in-vivo, Figs. 4–6, 8 also show CRLB, which indicated an improved reconstruction performance of DLTV. In Fig. 6 for example, metabolites were fitted with CRLB < 30% for Cr3.9(11%), Cr3.0(20%), Ch(4%), Spm(11%), Cit(6%) and Tau(28%) in DLTV, as opposed to TV which had only Cr3.9(14%), Ch(6%), Spm(11%) and Cit(5%), PM which had Cr3.0(20%), Ch(3%) and Spm(8%), and DL which had only Cr3.0(22%), Ch(10%) and Spm(24%). As expected, the results of DLTV showed reliable fit for metabolites which were individually picked up by either TV or DL.

DL builds a basis that can sparsely represent the data at hand as opposed to a fixed basis used by TV and PM, leading to a better sparse representation. However, the effectiveness of DL is also dependent on the quality of training data. The TV-filtered training data in DLTV, helps it to find a better sparse representation, leading to an overall improved performance. Similar improvement may be achieved by combining PM and DL as well [42, 62].

## Potential drawbacks and challenges of DLTV

One of the main drawbacks of DLTV is the increased reconstruction time due to learning dictionaries in each iteration. However, it has been reported that a graphics processing unit (GPU) can significantly improve this reconstruction time [63], which we have not used yet in this work. Another challenge with the implementation of DLTV is the additional reconstruction parameters that need to be tuned compared to using TV or DL independently. The effect of the choice of block size in DL, for example, is applicable in the case of DLTV as well. The larger size of blocks leads to over-smoothing, but retains noise if too small. A similar effect can be expected based on the choice of $\epsilon$ in eqn. [6]. While higher values of $\epsilon$ can introduce oversmoothing, lower values can lead to noise retention in both DL and DLTV. In addition to other parameters of DL like the choice of dictionary size and number of training samples, the strength of TV denoising can also affect the quality of reconstructed data in DLTV as it has a direct effect on the quality of training data.

It is observed that a set of parameters tuned for a particular undersampling factor using a phantom scan can be used for reconstructing in-vivo datasets at the same undersampling level. Choosing the parameters for in-vivo reconstruction based on phantom scans is, however, only approximate. A more accurate choice of parameters needs an estimation of sparsity level and the number of atoms in the dictionary, among other parameters such as size of the blocks used to train dictionaries for the data at hand, since these optimal values are data-dependent. The same is true for the optimal regularization parameters of other reconstruction techniques. However, the increased number of parameters puts DLTV at a disadvantage. In this work, we have shown the feasibility of applying dictionary learning for 5D EP-JRESI reconstructions, with parameters estimated from phantom scans to give performance on par or better than the less sophisticated reconstruction approaches. This approach is promising since further improvements can be achieved by using methods like adaptive sparsity level and dictionary size estimation for dictionary learning as reported in [64]**,** which is the future direction of the work. Further optimizations may be achieved by adjusting the strength of the TV filter in the DLTV reconstruction based on the feasibility of the piecewise constant assumption by first order gradient in TV and a priori information about the sparsity of data in the finite difference representation. Another limitation of this pilot study is the limited number of human PCa and healthy subjects.

## Conclusion

This work investigated the feasibility and performance of reconstructing undersampled 5D EP-JRESI data (8x and 12x) using multiple sparse representations within a CS framework. It is observed that the higher undersampling rates for MRSI can be made feasible with a more sophisticated reconstruction technique like hybrid DLTV, which considers the data to be sparse with respect to both a learned basis and in the finite difference-based representation. J-resolved MRSI is shown to be capable of reconstructing and clearly distinguishing Cit, Ch, Spm, mI, Glx, sI, Tau and Cr peaks as reported in ex vivo HR-MAS studies [59, 60]. 2D J-resolved spectroscopy combined with ProFit gives individual metabolite ratios as listed above in contrast with 1D spectroscopy where (Ch + Spm + Cr)/Cit is commonly used (61). While further optimization is needed to make the reconstruction more computationally efficient and more sensitive to metabolites at lower physiological concentrations, this approach can facilitate bringing down the total scan time of the 5D EP-JRESI scan from 21 to 14 min by using a 12x undersampling factor instead of 8x, assuming a TR of 1.2 s and 64 ${t}_{1}$ increments to encode the 2nd spectral dimension.

## Supplementary Information

Below is the link to the electronic supplementary material.

Supplementary file1 (DOCX 3942 KB)
