# Supplementary material for: Dictionary learning compressed sensing reconstruction: pilot validation of accelerated echo planar J-resolved spectroscopic imaging in prostate cancer
Source: MAGMA. 2022 Jul 23;35(4):667–82. doi: 10.1007/s10334-022-01029-z (PMC9363346; doi:10.1007/s10334-022-01029-z)

## SUPPORTING INFORMATION

### *CS reconstruction of 4D EP-J-Resolved Spectroscopic Imaging Acquired in Malignant PCa patients Using DLTV and TV*

***Patients:*** Twenty two PCa patients with a mean age of 63.8 years (range: 46–79 years) who subsequently underwent radical prostatectomy and a 72-year-old healthy male between March 2012 and May 2013, were selected for the study. Patients’ Gleason scores varied between 6 and 9. Prostate-specific antigen (PSA) levels ranged from 0.7 to 22.8 ng/mL (mean of 6.23 ng/mL). A 3T Siemens (Siemens Medical Solution, Erlangen, Germany) MRI scanner with an endorectal "receive" coil. The protocol combining MRI and MRS was performed at least 8 weeks after the transrectal ultrasound-guided sextant biopsy. The entire protocol was approved by the Institutional Review Board, and informed consent was obtained from each patient.

**Data Acquisition**

Since the fully sampled 4D EP-JRESI scan (TR of 1.5s, 16k_y_*32k_x_, 64t_1_, 512t_2_) can take more than 25 minutes, an 4X undersampled 4D EP-JRESI sequence was validated on the 3T MRI scanner. The volume of interest (VOI) was localized using three slice-selective radio-frequency (RF) pulses (90^0^-180^0^-180^0^) (29). The parameters for the EP-JRESI were: TR/TE = 1500/30ms, 2averages per encoding 16 phase encoding steps, 512 t_2_ complex points with an F_2_ bandwidth of 1190Hz. For the second dimension (F_1_), 64 increments with bandwidths of ±250Hz were used. The in plane spatial resolution was 1cm^3^.

**Results**

Shown in Fig. S1 are the reconstructed multi-voxel 2D J-resolved spectra using DLTV in a 72 years-old male subject. All the 2D spectra showed high Cit and peaks due to Ch/Spm (3.15ppm),/Cr (3ppm), mI (3.5ppm) and Cr3.9ppm very similar to that of healthy prostates. In this subject, the biopsy showed no malignancy and 3D MRSI was reflective of the same. The bottom panel in Fig. S2 shows multi-voxel 2D J-resolved spectra in a 72 years-old PCa patient (Gleason score of 3+3) in the base region of prostate, reconstructed using DLTV. Panels in the top row from left to right shows the MRSI VOI location and extracted 2D J-resolved spectrum using DLTV and TV. While biopsy showed malignancy in the left base (as well as left apex and mid), the 3D MRSI indicated malignancy in both left and right base. Multi-voxel 2D J-resolved spectra acquired in a 65 years-old PCa patient (Gleason score of 3+4) were reconstructed using DLTV as shown at the bottom of Fig. S3. The MRSI VOI location and extracted 2D J-resolved spectrum using DLTV and TV reconstructions are shown in the panels in the top row from left to right. The multi-voxel spectra showed elevated Ch and depleted Cit on the right apex/mid regions. Biopsy reported malignancy in the right apex which agreed with the 3D MRSI findings. Shown in Fig. S4 are expanded multi-voxel 2D J-resolved spectra reconstructed using DLTV from two MRSI VOI locations in a 63 years-old PCa patient (Gleason score of 7/8). Top and bottom panels represent VOI locations and multi-voxel spectra in the base and apex regions in the prostate, respectively. The results were in agreement with the biopsy reports that indicated malignancy in the right base and mid regions. These results demonstrate that, similar to the reconstruction of the undersampled 5D data, the DLTV reconstruction of the accelerated 4D EP-JRESI data shows superior quality compared to DL as well as gradient based TV and PM reconstructions.

**Fig.S1**. Multi-voxel 2D J-resolved spectra were reconstructed using DLTV from a 72-year-old male shown at the bottom and the MRSI VOI location is shown in the top row. Also shown are extracted 2D J-resolved spectrum reconstructed using DLTV and TV.


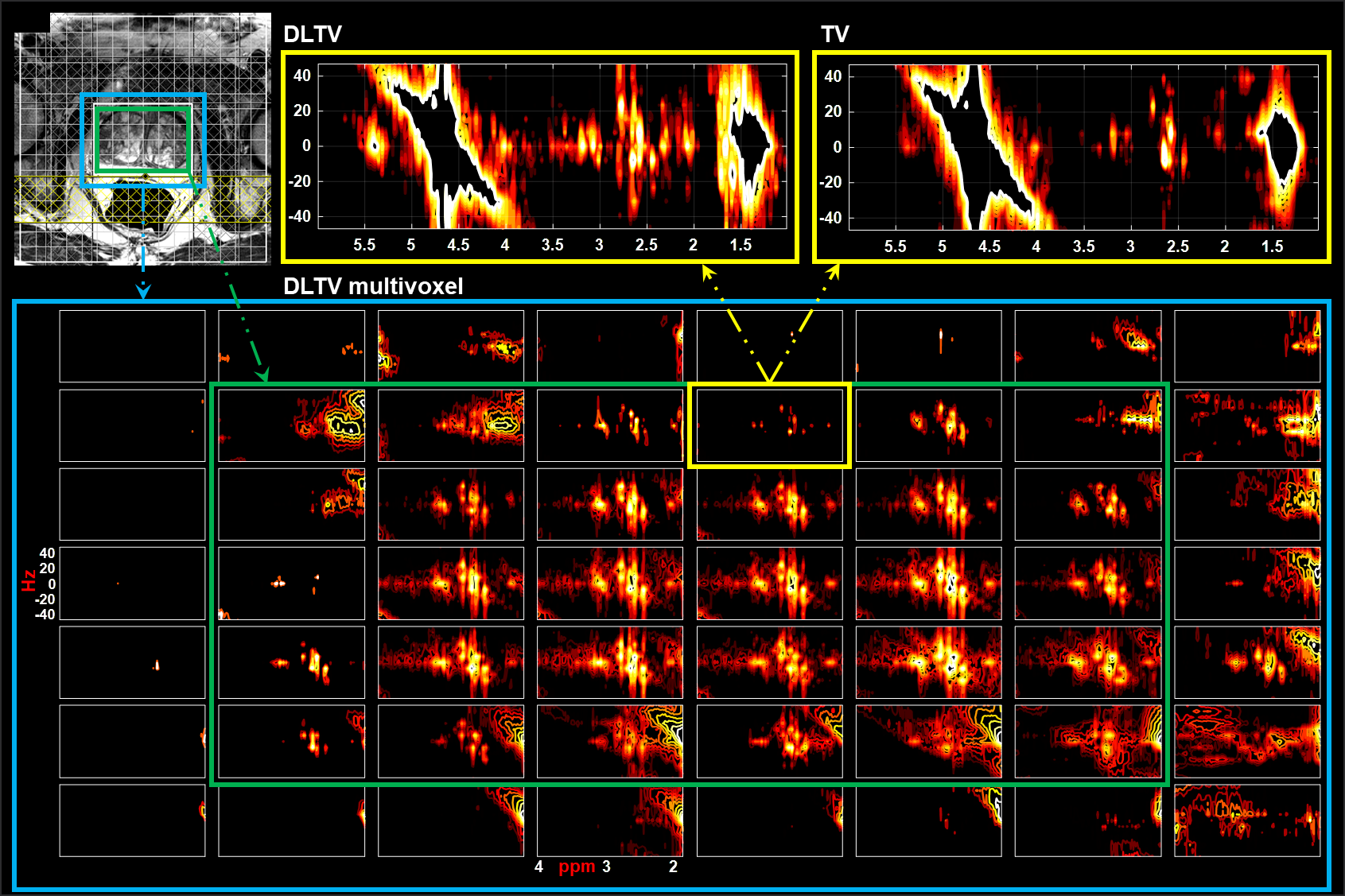


**Fig. S2**. (Bottom): Multi-voxel 2D J-resolved spectra were reconstructed using DLTV. (Top): The MRSI VOI location in a 72-year-old PCa patient is shown. Extracted 2D J-resolved spectrum using DLTV and TV is also shown.


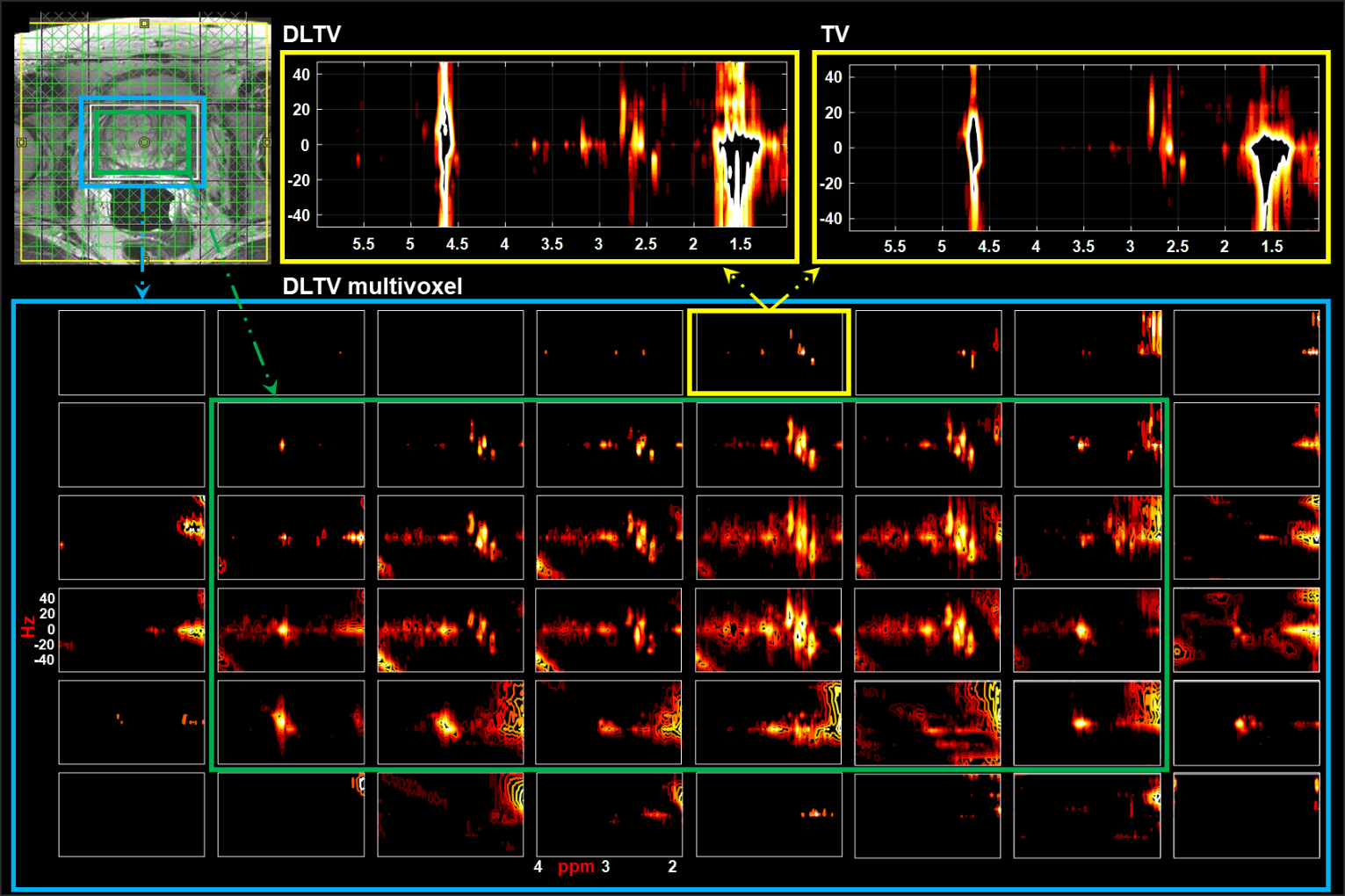


**Fig. S3**. Multi-voxel 2D J-resolved spectra were reconstructed using DLTV (Bottom). The MRSI VOI location in a 65-year-old PCa patient is shown in the top row. Extracted 2D J-resolved spectrum using DLTV and TV is also shown.


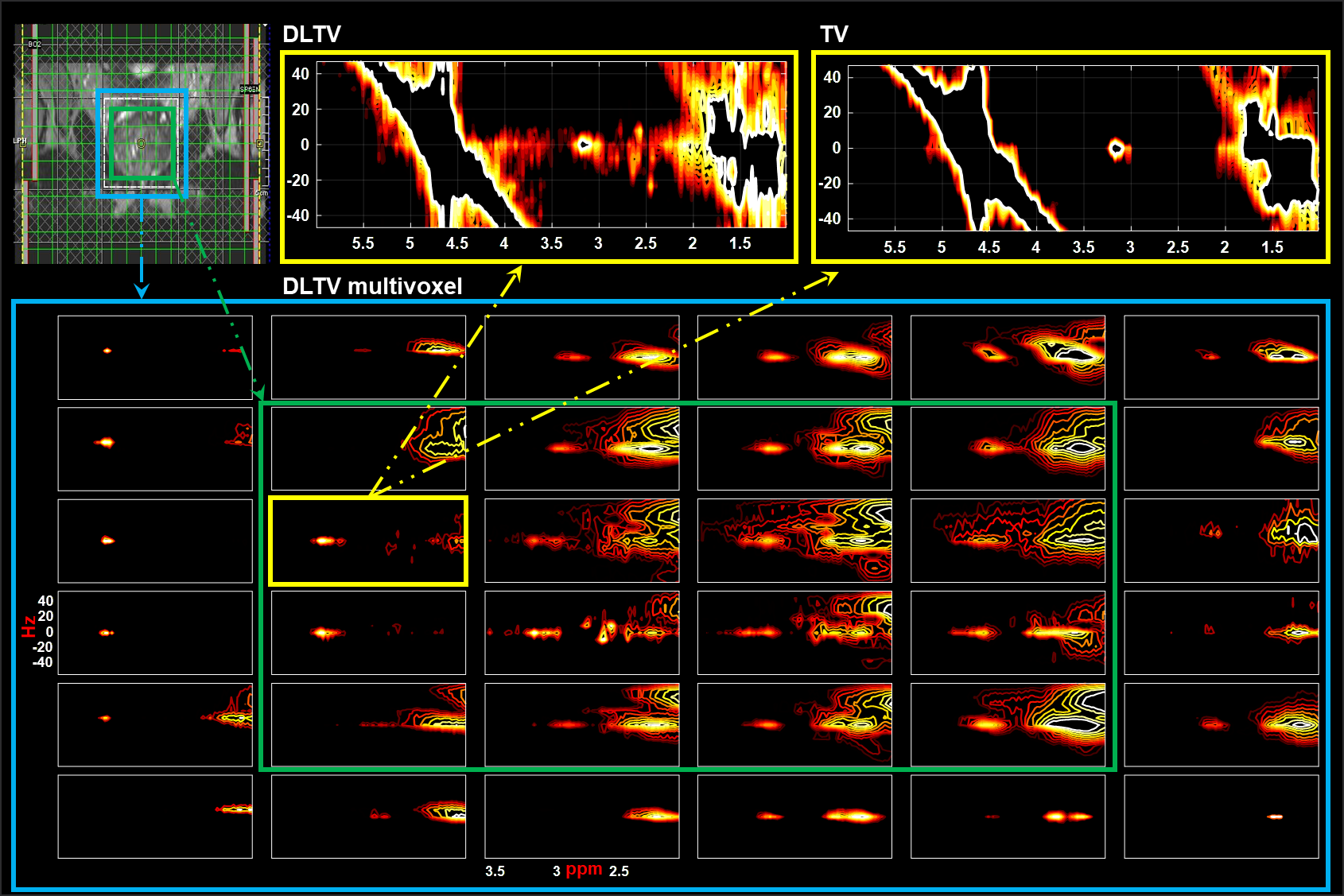


**Fig. S4.** Expanded multi-voxel 2D J-resolved spectra from two MRSI VOI locations in a 63-year-old PCa patient were reconstructed using DLTV.


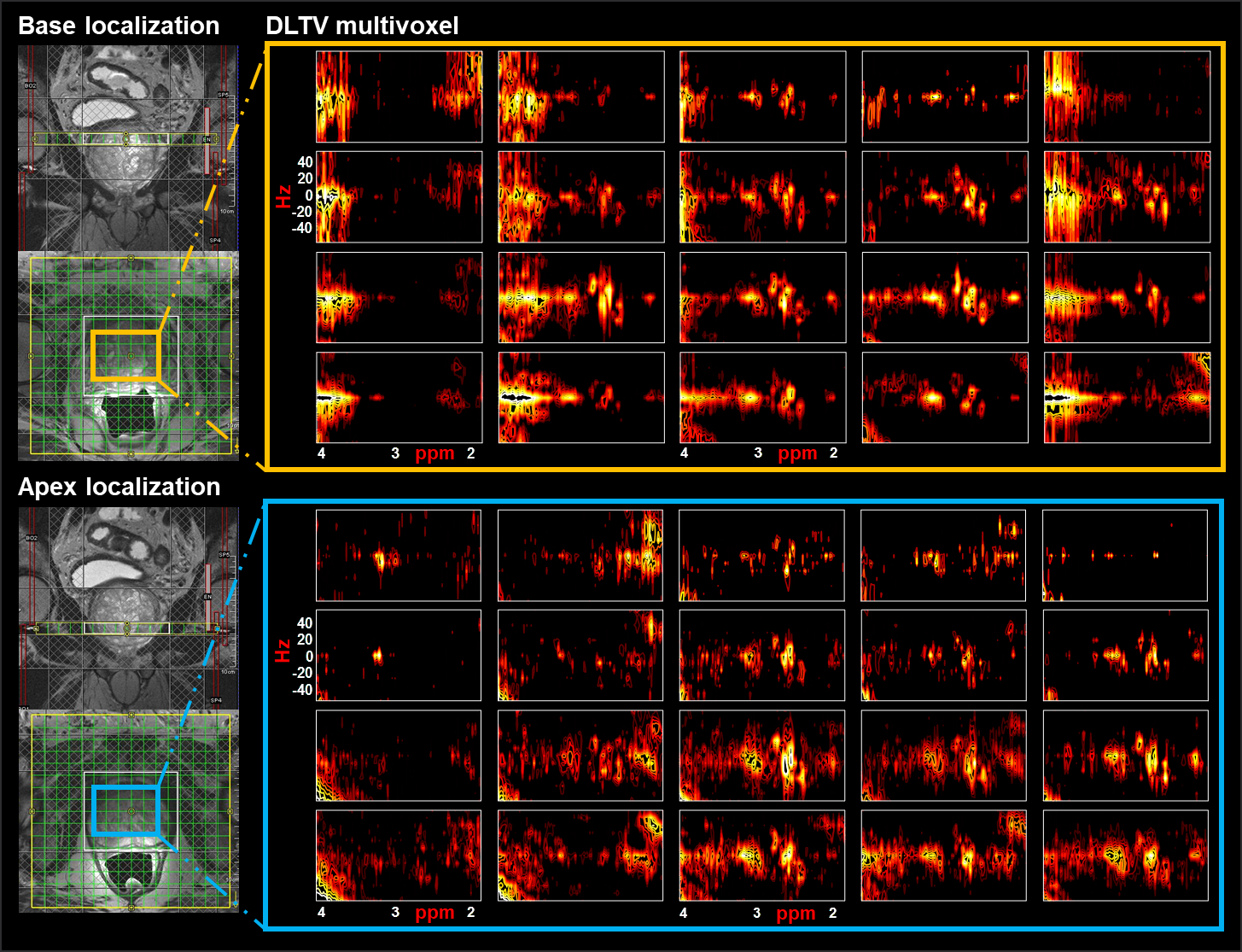

Supplement: Supplementary file 1 — Supplementary file1 (DOCX 3942 KB) [file 10334_2022_1029_MOESM1_ESM.docx]
